# High-speed microscopy of continuously moving cell culture vessels

**DOI:** 10.1038/srep34038

**Published:** 2016-09-26

**Authors:** Friedrich Walter Schenk, Nicolai Brill, Ulrich Marx, Daniel Hardt, Niels König, Robert Schmitt

**Affiliations:** 1 Fraunhofer Institute for Production Technology IPT, Department of Production Metrology, Aachen, 52074, Germany; 2 Mabri. Vision GmbH, Aachen, 52068, Germany; 3 Laboratory for Machine Tools and Production Engineering (WZL) of RWTH Aachen University, Aachen, 52074, Germany

## Abstract

We report a method of high-speed phase contrast and bright field microscopy which permits large cell culture vessels to be scanned at much higher speed (up to 30 times faster) than when conventional methods are used without compromising image quality. The object under investigation moves continuously and is captured using a flash illumination which creates an exposure time short enough to prevent motion blur. During the scan the object always stays in focus due to a novel hardware-autofocus system.

Due to the microscope’s small field of view (depending mainly on the objective lens’s magnification) an enormous number of single images have to be taken in order to image large objects, like whole microtiter plates (MTPs). Conventional automated microscopes use an incremental scanning routine to perform this task. This involves repeatedly positioning the object, adjusting the focus and capturing a segment of the stationary object. With thousands of image positions on an MTP this is a very time-consuming process.

The imaging rate can be increased dramatically by omitting to stop for each acquisition and imaging the object during a continuous movement. This acquisition method, termed continuous scanning, can be used in conjunction with line and area scan cameras and was first reported in the literature a few decades ago^1^,^2^. The main challenges related to this acquisition concept are the need to prevent motion blur and to ensure that the object is kept continually in focus.

## Results

### Concept

**Figure 1:**
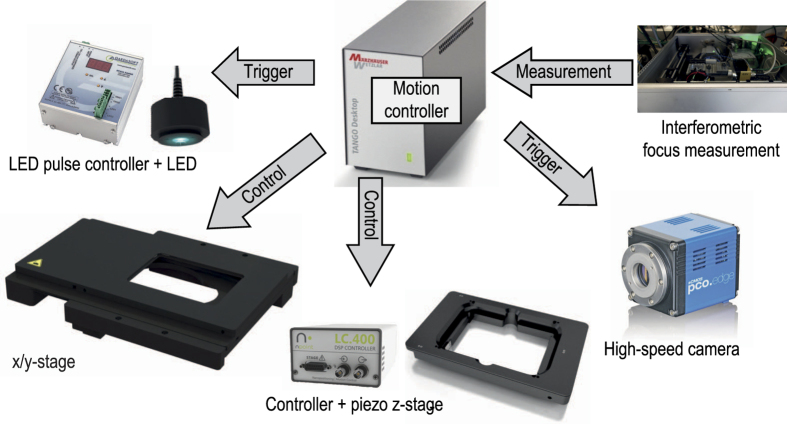
Special hardware components necessary to upgrade a conventional microscope to a high-speed scanning system featuring image acquisition of moving objects (permission to publish the product photos is kindly granted by the manufacturers Märzhäuser, Gardasoft and PCO).

**Figure 2:**
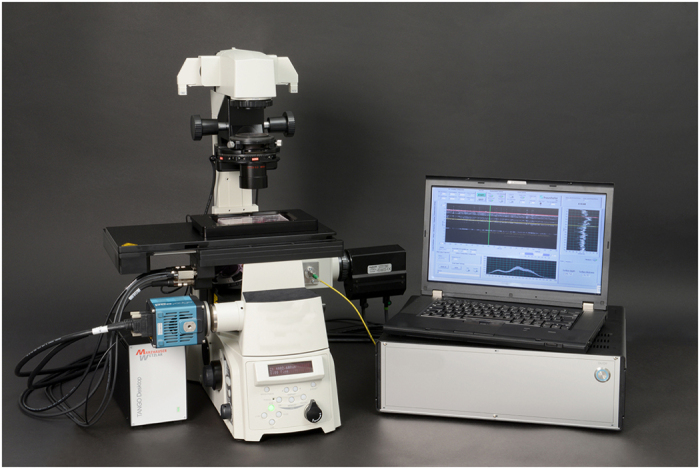
Implementation of the high-speed microscopy solution with the OCT-based autofocus system on an inverted Nikon Ti-E microscope. The fibre of the focus measurement system is coupled into the microscope using a modification on the fluorescence port. See also the dotted line in the drawing of Fig. 4a. Coupling via the camera port is shown in Fig. 4b.

We have developed a high-speed microscopy solution using the latest hard- and software technology and a novel autofocus technique that fulfils both requirements (Figs 1 and 2).

Equipped with a 5.5 megapixel sCMOS camera, frame rates of up to 98 full frame images per second are achievable, which greatly reduces the duration of the imaging process. To keep the frame rate high most of the time, the number of stops is reduced to a minimum by scanning in a meander-like motion along the whole MTP instead of imaging it well by well. This way, only two stops per line are necessary. For a whole microtiter plate, the scanning time is reduced to approximately 10%, 4% or 3% when using a 4x, 10x or 20x objective compared to the conventional stop-and-go scanning process implemented on the same microscope (Nikon Ti-E) (see Supplementary Table S1).

The fastest slide scanner of the 2^nd^ International Scanner Contest (ISC), the latest ISC organized by the Charité in Berlin in 2012, was the model Pannoramic 250 from 3D Histech. It achieved a throughput of 0.86 cm^2^/min for HE stained slides in combination with a 20x objective^3^. The focus corrected speed of this winning scanner with a 40x objective was 0.62 cm^2^/min. The continuous high-speed microscopy approach presented in this paper achieves a throughput of 7.84 cm^2^/min (quadratic area of 2.8 cm × 2.8 cm in one minute) with a 20x objective and 2.25 cm^2^/min (quadratic area of 1.5 cm × 1.5 cm) with a 40x objective using the movement parameters given under Supplementary Table S1. This is more than 9.1 times respectively 3.6 times faster (20x resp. 40x objective) compared to the fastest commercially available slide scanner tested at 2^nd^ International Scanner Contest.

In transmitted light microscopy modes like phase contrast and brightfield imaging, the exposure time can be limited to a few microseconds using an intense LED flash^4^ to achieve motion blur free images with adequate signal-to-noise ratio^5^ (Supplementary Table S2). The use of TDI (time delay and integration) cameras is essential in order to capture enough emission light of moving fluorescence microscopy samples^6^. Equipped with a TDI camera instead of a conventional matrix camera and a strong fluorescence light source the continuous scanning approach would also work for fluorescence microscopy samples. However, this is not within the scope of this article that only deals with a high-speed microscopy solution for transmitted light phase contrast and brightfield microscopy.

### Autofocus for continuous scanning

In the case of continuously moving samples, the autofocusing procedure is more difficult as the time to measure and adjust the focus distance is very short. Conventional software autofocus methods require a stationary object which is imaged at different heights (z-stack generation) to determine the focus distance based on the sharpest image^7^. As this is time-consuming it can only be used to determine the focus position from a few sampling points combined with interpolations to create a focus map^8^ beforehand. This, however, can introduce focus errors as the bottom of plastic microtiter plates is quite uneven. Several methods based on the simultaneous or accelerated capture of the different layers of the z-stack have been developed in order to speed up the software autofocus procedure^9^,^10^,^11^. However, these modifications to the classical software autofocus approach do not work properly when there is a large deviation of the focal plane as in the case of microtiter plates (a few hundred microns).

Apart from software-based autofocus methods that rely on the evaluation of images for focus determination, hardware-based autofocus measurements have become popular in cell biology applications^12^. These systems are based on the reflection of a laser beam at the interfaces of the substrate. Commercially available laser autofocus systems like Nikon’s *Perfect Focus System* or Olympus’ *ZDC* which are intended for focus drift compensation are limited in their measurement capability as they can resolve only the first reflecting surface of a sample. In the case of microtiter plates on an inverse microscope, this corresponds to the underside of the plate bottom. The interesting layer where the adherent cells are located, however, is the upper side of the plate bottom (or slightly above) that cannot be measured by these systems. As the plate bottom thickness varies strongly (up to 300 μm across one plate) due to the injection moulding manufacturing process, adding a fixed offset to the detected underside does not yield the focus plane required. What is needed, is a system that can directly measure the upper side of the plate bottom.

**Figure 3:**
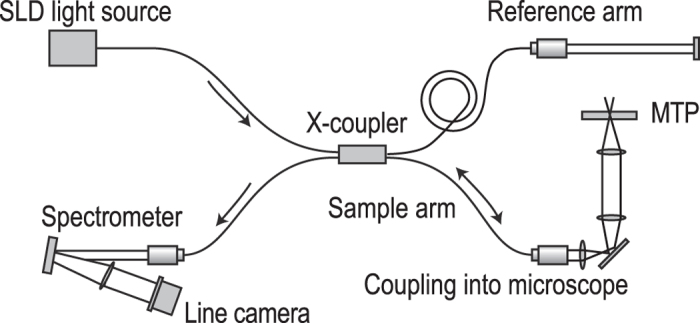
Schematic representation of the focus measurement system based on a fiber-coupled Michelson interferometer design. Light coming from the superluminescent diode (SLD) is split into a reference and a measurement arm and is reflected from a fixed reference mirror and from the various interfaces of the object (microtiter plate - MTP). The reflected light portions are combined in the spectrometer arm where they interfere with each other. The interference pattern is captured by an optical spectrometer.

For this purpose, we have developed an interferometric focus measurement system based on Fourier domain optical coherence tomography (FD-OCT)^13^ (Fig. 3).

**Figure 4:**
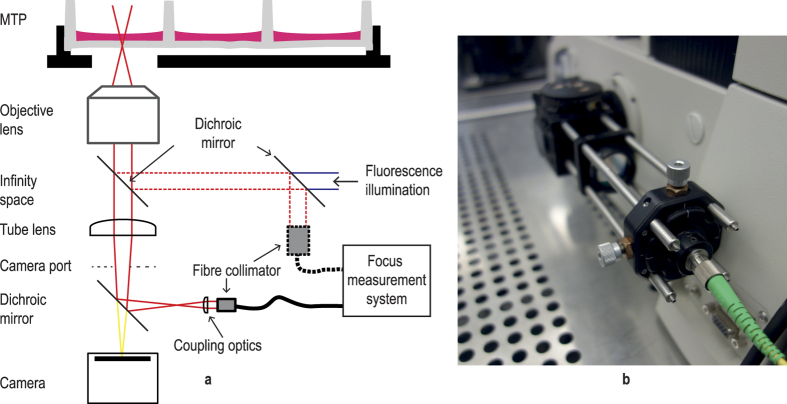
Options for coupling the focus measurement beam into the microscope. (**a**) Schematic, (**b**) photo of modified camera side port. Note that the imaging camera is mounted to another camera port of the microscope on the photo.

It measures the focus position several thousand times per second with no moving parts thereby allowing the object to move uninterruptedly during measurement. A near-infrared laser beam at 840 nm is coupled into the microscope through a beam splitter in front of the C-mount camera port allowing synchronous focus measurements and imaging of the moving object (Fig. 4).

**Figure 5:**
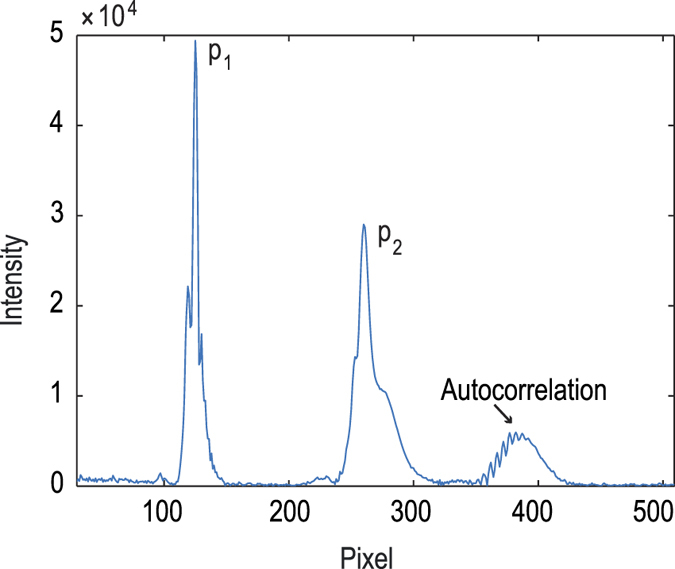
Spectrum (a-scan) of the focus measurement after applying a fast Fourier transform (FFT) to the interference raw signal. The lower (p_1_) and upper (p_2_) reflection peaks from the microtiter plate bottom are clearly visible. There is another peak formed through the autocorrelation of the two plate bottom reflections which resides around pixel position 390.

In contrast to commercially available laser-based focus measurement systems, our system is able to resolve multiple interfaces of the measured object simultaneously. Each reflection from an interface of the sample leads to a peak in the spectrum (Fig. 5, peak 1 = plate underside, interface air-plastic, peak 2 = plate upper side, interface plastic-medium) that is determined at subpixel resolution with a peak fit algorithm (Methods section).

The geometrical position of the focal plane, the upper side of the plate bottom, can be calculated on the basis of the peak positions, which decode the optical path length differences between the interfaces of the sample and a fixed reference mirror (Methods section).

**Figure 6:**
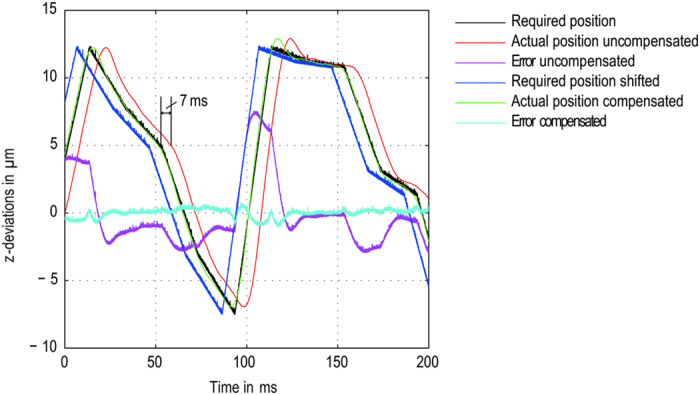
Compensation of the delay between required and actual position. The reaction time for the piezo z-stage (nPoint 300 μm) to reach the required position is about 7 ms for a random height profile. By shifting the required positions by the product of the reaction time and the scan velocity, the focus error (required z-position minus actual z-position) can be greatly reduced below the depth of field.

For fast scanning applications of microtiter plates, it proved useful to carry out a quick, continuous focus scan at the stage’s maximum velocity using the OCT-based focus sensor before the actual imaging scan. In this way the resulting focus values can be filtered, especially at the edges of or between the wells where no valid measurement data is obtained. The high measurement rate permits thousands of focus sampling points to be measured. Averaging these data points attenuates the effect of outliers and results in robust focus values for each field of view. Additionally, the focus correction procedure can be optimized when all focus values are known before the continuous imaging scan is conducted. In our solution we use a highly dynamic piezo z-stage to synchronize the z-axis position with the movement in scan direction. The misregistration that would occur due to the fast lateral object movement and the lapse of time that it takes for the piezo to reach its required positon can be easily compensated by relocating the focal plane positions relative to the scan direction (Fig. 6).

### Image preprocessing

**Figure 7:**
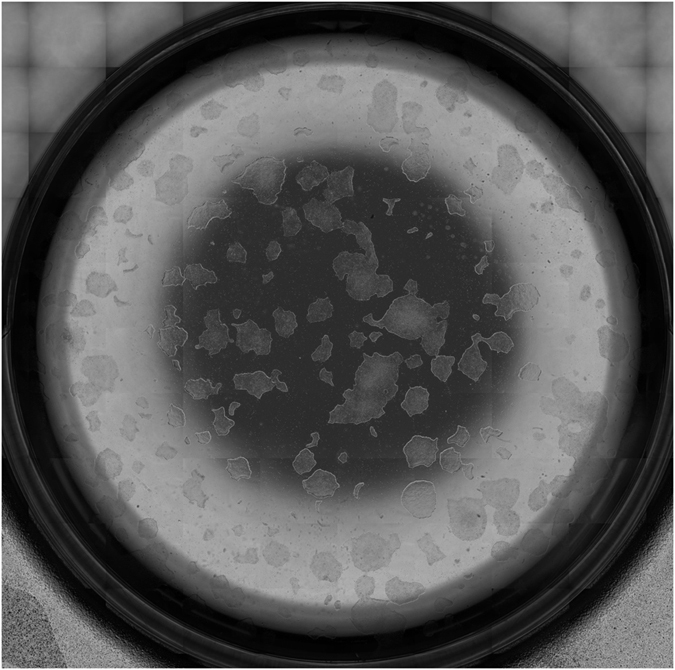
Stitched image of continuous imaging scan of one well of a 6-well microtiter plate filled with adherent iPS cell colonies on Matrigel cultivated in mTeSR. Magnification: 4x, imaging mode: phase contrast, scan velocity: 34 mm/s, pre-processing: shading correction.

Besides creating single images with uncompromised quality that are free of motion blur due to the short flash exposure and adequately focused by virtue of the novel hardware autofocus system, our solution provides excellent image mosaics to display large parts of the sample at high resolution (Fig. 7). We achieve this by using a fast stitching approach (Methods section). Other preprocessing steps to enhance the image quality include a camera calibration, shading correction and histogram normalization.

## Discussion

The new high speed imaging solution is one of the methods used within the StemCellFactory (www.stemcellfactory.de), a fully automated production unit for the automated generation of induced pluripotent stem cells (iPS cells)^14^. The new high-speed microscopy system permits hundreds of MTPs to be examined every day. The solution provides fast imaging of the entire well content with microscopic resolution to automatically assess the confluence and status of the cell culture. This is crucial for the automated quality and process control of the unit^15^.

The high-speed microscopy acquisition method based on a continuous object movement can be applied easily to most microscopes on the market. The cost of such a speed upgrade for conventional microscopes is quite low. Apart from cost-efficient commercially available components, the interferometric focus measurement sensor is the only in-house-developed hardware unit with material costs around 10.000 Euros (Supplementary Table S3).

The high-speed microscopy approach is not only useful for fast imaging of complete microtiter plates but can speed up microscopic inspections in other fields like industrial quality control as well.

## Methods

### Components

**Figure 8:**
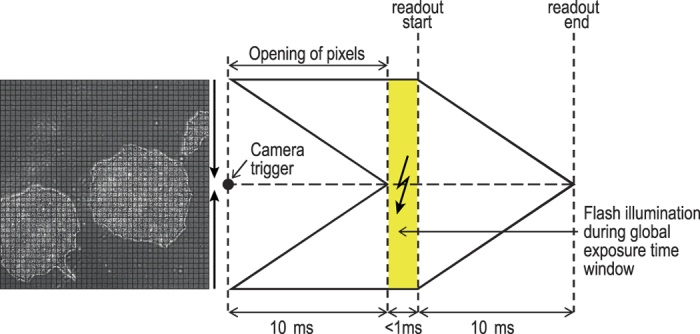
Flash illumination during global exposure time window when all pixels are opened thus avoiding the rolling shutter effect.

The high-speed microscopy solution, which is based on imaging continuously moving objects, is implemented on an inverse research microscope (Nikon Ti-E). We use it mainly in conjunction with phase contrast objectives with 4x, 10x and 20x optical magnification. Higher magnifications and other contrast modalities like differential interference contrast (DIC) can also be used. The core component of the high-speed microscopy solution developed is a TANGO 4 stage controller from Märzhäuser Wetzlar. It ensures hardware-based synchronization of the components and is operated with a firmware that has been enhanced to provide all necessary functionality. It controls both the SCANplus IM 130 × 85 (also Märzhäuser Wetzlar) scanning stage which has an integrated measuring system and the 300 μm piezo z-stage via analogue output to the piezo controller LC.400 (both from nPoint). It also creates the TTL trigger impulses whose purpose is to synchronize the stage position with the high-speed camera pco.edge 5.5 (pco) and the flash controller RT220F-20 (Gardasoft) which controls an LED based transmitted light illumination (LED 100 from Märzhäuser). It is equipped with a Cree XR-E high-power LED. Flash durations as short as 1 μs can be generated when the illumination source is attached to the Gardasoft controller. To generate enough photons for sufficient exposure, the LED brightness can be increased by driving it with more than the current rating for short pulses (up to tenfold overdriving). In phase contrast microscopy, a significant proportion of the light is blocked by the condenser annulus. In addition to a bright illumination source, a sensitive camera is therefore crucial. The sCMOS camera used, combines sensitivity with fast frame readouts and therefore ideally matches the requirements for the flash based high-speed microscopy solution. It is operated in the rolling shutter mode which offers the lowest readout noise. The rolling shutter effect is avoided^16^ by shifting the flash illumination to the global exposure time window when all pixel rows are active (10 ms after the exposure trigger signal) (Fig. 8).

A measurement system developed in-house was built for fast focus measuring operations. The working principle of this hardware-based focus measurement system is the same as in spectral domain (SD) optical coherence tomography (OCT). The system is based on a fibre-coupled Michelson interferometer design (Fig. 3) and operates at a wavelength range around 840 nm, which does not affect the microscopic image. The light source is a superluminescent diode (Superlum SLD-351-HP) with a spectral width of 60 nm. The core of the system is a high-resolution spectrometer which decodes the spectrally encoded depth information. The spectrometer optics system was designed specifically for this task. The maximum measurement rate of the spectrometer line camera used (e2v AViiVA EM1 GigE) is 78 kHz at a bit depth of 12 bit. The line sensor has 1024 pixels, achieving a theoretical measurement range of about 2.9 mm. This is sufficient to capture both peaks from the lower and upper bottom of a microtiter plate reliably (Fig. 5).

The near-infrared measurement beam can be coupled into the microscope in two ways using dichroic mirrors. This can be achieved either by modifying the epi-fluorescence light port (see Fig. 2) or via one of the camera ports (Fig. 4). When leaving the fibre, the measurement beam is first collimated by a fibre collimator (Thorlabs F220APC-780). Then it is focused into the focal point of the tube lens of the microscope (the intermediate image plane) by a converging lens. As a consequence, the measurement rays become collimated in the parallel optical path between tube and objective lens. They are focused onto the object where they are reflected at the various sample interfaces. On their way back to the spectrometer the reflected portions of the light from the sample interfere with the light reflected from the reference mirror. The spectrally encoded depth information is analysed by the spectrometer. A complete mathematical derivation of the working principle is provided by Izat *et al.*^17^ After applying a fast Fourier transform (FFT) to the interference raw signal of the spectrometer line camera, a spectrum (called a-scan) is generated (Fig. 5).

### Calculation of focal plane

Each reflection from the sample leads to a Gaussian shaped peak in the spectrum. In order to precisely calculate the focal plane of the object, it is necessary to detect the exact peak positions with subpixel resolution. For this purpose we use Caruana’s peak fit algorithm^18^. It is applied to the two sections of the spectrum where the peaks from the lower and upper plate bottom reside and calculates the peak positions for peaks whose amplitudes exceed a certain threshold thus avoiding the detection of noise peaks.

The peak positions indicate the optical path length differences between the reflection from the sample and the fixed reference mirror. With this information it is possible to calculate the absolute geometrical position of the upper plate bottom, hence the focal plane to which the adherent cells are attached.

The geometrical position *z*_1_ of the first reflection is obtained by multiplying the peak position *p*_1_ with a constant proportionality factor *K* plus an additional offset *z*_Offset_


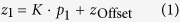


**Figure 9:**
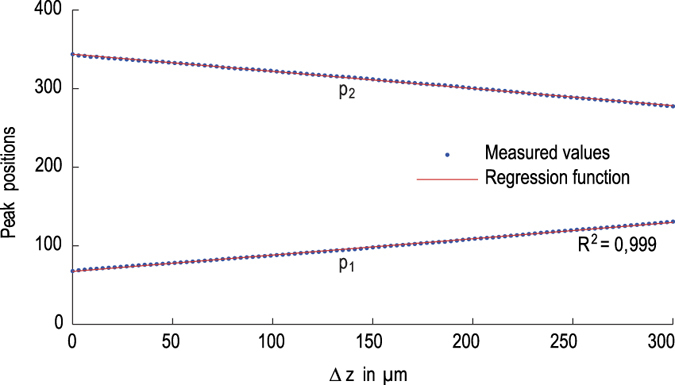
Linearity of the peak positions when the object is evenly elevated by the piezo stage from 0 to 300 μm. Note that the peak positions travel in opposite direction. This is because one of the peaks was deliberately mirrored by setting the length of the reference arm accordingly. The advantage of this is that the peak positions can be easily separated and do not extend into each other’s range which facilitates the peak determination. Furthermore the range of the two peaks can be controlled in order not to overlap with the autocorrelation peak visible in Fig. 5.

The factor *K* can be constant as there is a linear relationship between the peak and the geometrical position of the reflection. The value of *K* corresponds to the inverse slope of the straight line which is generated when plotting the peak positions over the geometrical shift of the object in axial direction preferably by using the calibrated piezo z-stage (Fig. 9).

The geometrical position of the second peak is influenced by the thickness of the plate bottom which is made of optically denser plastic material (*n*_MTP_ ~ 1, 5) that the light beam has to pass through. To compensate for this, the difference between the two peak positions has to be divided by the refractive index *n*_MTP_ of the plastic material which is constant for the whole plate. Thus *z*_2_ can be calculated as


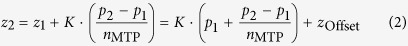


In order to calculate the z-positions at different locations of the plate, it is sufficient to focus the system only once manually or with a software autofocus procedure onto *z*_2,focus_ to know the offset *z*_Offset_.

Any new focus position *z*_2,new_ can be calculated by *z*_2,new_ = *z*_2,focus_ + *δz*_2_ where *δz*_2_ is the relative deviation of the focal plane which is given by


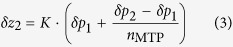


In this formula *δp1* respectively *δp2* indicate the changes of the peak positions compared to the focused state at *z2*, focus.

### Microscope control soft- and hardware

All microscope components are controlled by a C++ program developed in-house. It features modular, object-oriented architecture. The software makes extensive use of multithreading technology by using Intel’s Threading Building Blocks (TBB) template library. To be compatible with a wide range of hardware components we use the driver layer of the open-source microscope control software μManager^19^. Computationally intensive tasks, especially in the image preprocessing, are GPU-accelerated using mainly the open-source library OpenCV^20^. In terms of hardware we use a powerful workstation with 192 GB of random-access memory (RAM) in order to buffer and efficiently process the large amounts of incoming imaging data.

### Stitching

**Figure 10:**
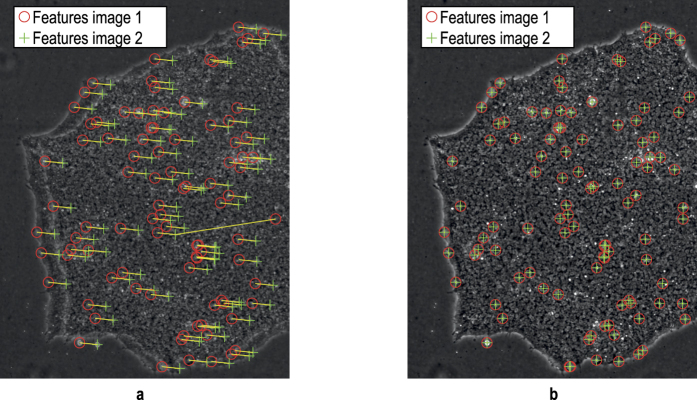
Feature detection using SURF keypoint descriptors in the overlapping image region. (**a**) Translation between images of (40, −4) pixels. (**b**) Alignment of both images after compensation of detected translation.

The response time of the motion control and trigger generation based on the stage positions, is 40 μs with the motion controller used. This allows the fields of view on the object to be captured quite precisely but not with perfectly equidistant spacing. The latency of the trigger signal leads to stochastic variations in the position of the field of view up to 40 μs times the scan velocity. To correct for this, we implemented a fast stitching procedure. Neighbouring fields are captured with a slight overlap and the relative position to each other is detected using GPU accelerated feature detection based on the SURF (speeded up robust features) algorithm^21^ (Fig. 10).

**Figure 11:**
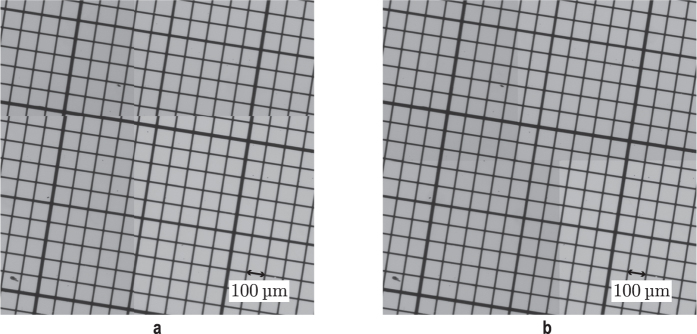
Software-based stitching generating seamless scanning result. To illustrate the benefit of using our stitching approach, we imaged a tilted calibration grid with a line distance of 100 μm at a scanning speed of 60 mm/s using a 4x objective. The result on the left (**a**) without overlap in the image acquisition and without a post image alignment exhibits visible transitions whereas application of the stitching method ensures an exact fit of all image data (**b**).

For performance reasons we compute the feature detection on a graphics card (Nvidia GTX Titan X). With calibrated image data^22^ there is only a varying translation between neighbouring images that has to be detected in order to correctly align each tile in the image mosaic. Occasional outliers can be filtered out by comparing each translation with the expected range based on the trigger and positioning uncertainty (40 μs*scan velocity). Instead of merely aligning each image with its predecessor we use global optimization inspired by Steckhan *et al.*^23^ which takes into consideration all surrounding images in a 4-connected neighbourhood. In a weighted least-squares approach, a sparse overdetermined linear equation system is formed with the goal of minimizing the global alignment error (Fig. 11).

### Further image preprocessing

**Figure 12:**
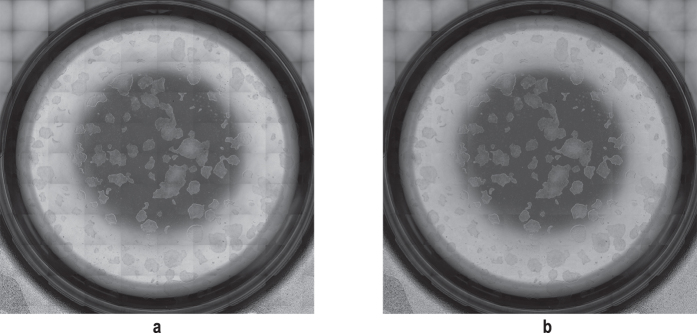
Benefit of shading correction in rendering the single tiles almost invisible. (**a**) Without shading correction, (**b**) with shading correction.

Apart from software-based image stitching to enhance the accuracy of the alignment we apply a shading correction algorithm to compensate for illumination inhomogeneities on each image tile^24^. This improves the overall appearance of the image as it renders the single tiles almost invisible as can be seen when comparing a stitched image of one well with and without shading correction (Fig. 12).

In order to improve the image contrast, we apply a histogram normalization combined with a bit depth reduction to the image data. The interesting part of the histogram of the original 16 bit raw data is displayed on an 8 bit range by linearly scaling all images.

### Stem cell culture

Induced pluripotent stem cells (iPS cells)^25^ are created by reprogramming somatic cells via targeted delivery of a specific set of reprogramming factors. These include the transcription factors Oct4, Sox2, c-Myc and Klf4. After 25 days of cultivation, emerging iPS cells are isolated by mechanically picking individual colonies, which are further expanded under specific culture conditions with the growth factor bFGF.

## Additional Information

**How to cite this article**: Schenk, F. W. *et al.* High-speed microscopy of continuously moving cell culture vessels. *Sci. Rep.* **6**, 34038; doi: 10.1038/srep34038 (2016).

## Supplementary Material

Supplementary Information
