# Supplementary material for: High-speed microscopy of continuously moving cell culture vessels
Source: Sci Rep. 2016 Sep 26;6:34038. doi: 10.1038/srep34038 (PMC5036042; doi:10.1038/srep34038)
Supplement: Supplementary Information [file srep34038-s1.pdf]

High-speed microscopy of continuously moving cell culture vessels

Friedrich Walter Schenk, Nicolai Brill, Ulrich Marx, Daniel Hardt, Niels König, Robert Schmitt

Supplementary Table S1: Scanning time calculations

Objective	Images ($x \times y$)	**Incremental** Duration *h:mm:ss*	**Incremental** Frame rate ∅	**Continuous** Duration *h:mm:ss*	**Continuous** Frame rate ∅	Temporal percentage cont./incr.
4x	665 (35×19)	0:08:06	1,4	0:00:46	15	10 %
10x	4042 (86×47)	0:43:31	1,5	0:01:57	37	4 %
20x	16 074 (171×94)	2:41:37	1,7	0:04:55	74	3 %

The basis for calculation is the relevant culturing area of a full microtiter plate of $114 \times 74\,\mathrm{mm}^2$. The scan direction is along the longer dimension of the object (x-axis). Each scan line is extended by 5 mm on both sides to provide an acceleration section. The entire sCMOS sensor with 2560×2160 pixels is used whereby it is advantageous to align the camera sensor so that the longer side of the sensor is parallel to the y-axis which results in fewer scan lines. For the stitching algorithm, an overlap of 5 % is used. The maximum readout rate of the camera sensor is 98 fps and the maximum scan velocity of the x/y-stage is 120 mm/s. The acceleration of the stage is set to $0{,}25\,\mathrm{m/s}^2$. After each stop a pause time of 0,5 s is maintained to let the oscillations of the liquid culture medium decay to a moderate degree.

Supplementary Table S2: Maximum exposure time for an image without noticeable motion blur[1]

$t_{\exp_{\max}}$		Velocity in mm/s			
		10	40	80	120
Objective	4x	162,5 µs	40,6 µs	20,3 µs	13,5 µs
	10x	65,0 µs	16,3 µs	8,1 µs	5,4 µs
	20x	44,7 µs	11,2 µs	5,6 µs	-

The values for $t_{\exp_{\max}}$ are calculated according to

$$t_{\exp_{\max}} = \frac{l_{\text{blur tolerance}}}{v_{\text{Object}} \cdot M} \qquad \text{with } l_{\text{blur tolerance}} = \max\left(l_{\text{Pixel}}, \frac{D}{2} \cdot M\right) \tag{1}$$

where M is the magnification factor of the objective lens and D is the lateral resolution limit defined by

$$D = \frac{1.22\lambda}{\text{NA}_{\text{Condenser}} + \text{NA}_{\text{Objective}}}. \tag{2}$$

[1]Blur tolerance: Projected movement on detector $\leq$ 1 pixel or half the lateral resolution limit times magnification factor.

Supplementary Table S3: Main components for the high-speed microscopy solution and associated costs

Component	Manufacturer and type	Price
Stage controller	Märzhäuser TANGO 4 plus Aux I/O option	2500 €
Motorized stage	Märzhäuser SCANplus IM 130 × 85	6600 €
LED pulse controller	Gardasoft RT220F-20	1100 €
LED illumination	Märzhäuser LED 100	650 €
High-speed camera	PCO pco.edge 5.5	12 000 €
Piezo z-stage with controller	nPoint Z300 with LC.400	8500 €
Interferometric focus measurement	Developed and manufactured in-house	10 000 €[2]

[2]Material costs.
